# Telomeres, DNA Damage and Ageing: Potential Leads from Ayurvedic Rasayana (Anti-Ageing) Drugs

**DOI:** 10.3390/jcm9082544

**Published:** 2020-08-06

**Authors:** Rohit Sharma, Natália Martins

**Affiliations:** 1 Department of Rasashastra and Bhaishajya Kalpana, Faculty of Ayurveda, Institute of Medical Sciences, Banaras Hindu University, Varanasi, Uttar Pradesh 221005, India; 2 Faculty of Medicine, University of Porto, Alameda Prof. Hernani Monteiro, 4200-319 Porto, Portugal; 3 Institute for research and Innovation in Health (i3S), University of Porto, Rua Alfredo Allen, 4200-135 Porto, Portugal; 4 Laboratory of Neuropsychophysiology, Faculty of Psychology and Education Sciences, University of Porto, 4200-319 Porto, Portugal

Ageing, while a relentless, unidirectional and pleiotropic phenomenon of life, is a key trigger for several age-related disorders, such as cancer, cataract, osteoporosis, hypertension, cardiovascular (CV), metabolic and even neurodegenerative ailments, including Alzheimer’s (AD) and Parkinson’s (PD) disease [1]. Telomeres shortening has been pointed to as the main factor that speeds up cell ageing and promotes degeneration processes [2]. With each DNA replication, the telomeres are progressively shortened, leading to the appearance of critically shorter telomeres. Telomerase is the key enzyme involved in the chromosomes (telomeres) ends protection and repair from shortening (adding repetitions of TTAGGG) during replication, consequently preventing catastrophic DNA loss and promoting the maintenance of healthy cell function [3]. However, telomerase activity is very low in human cells, and thus, low telomerase activity, leads to the imminent appearance of short telomeres and to a low rate of DNA repair, consequently promoting an accelerated ageing process [4,5,6]. Briefly, the main sources of telomere shortening or DNA damage can be (i) exogenous, such as radiation, unhealthy diet and lifestyle, mental stress and environmental chemicals, or (ii) endogenous, such as chronic inflammation, chemical instability (purification), spontaneous errors during DNA replication and repair and oxidative stress [7]. Quite recently, and owing to limited efficacy of conventional drugs as anti-ageing modulators, options are being searched from natural products and traditional medicines with potential to arrest or delay ageing.

Ayurvedic medicines, having historical roots more than 5000 years ago, have been increasingly searched for worldwide for multiple purposes. For instance, several Ayurvedic medicinal herbs and formulations, traditionally known as *Rasayana*, have been shown to markedly promote health, immunity, vigor, vitality, and longevity, at same time as protecting from stress. These medicines claim to facilitate healthy ageing, arrest degenerative changes and have rejuvenating potential at cell and tissue levels [8,9]. In this sense, here we briefly discuss the evidence-based perspectives of some of these anti-ageing drugs, considering their role in promoting telomerase activity, telomere length and DNA repair.

There are some Ayurvedic *Rasayana* herbs and formulations with potential telomer protective and DNA repair activities (Figure 1).

Ashwagandha [*Withania somnifera* (L.) Dunal], aka Indian ginseng, is a flagship rejuvenating and adaptogen Ayurvedic herb, traditionally used as an anti-ageing agent. Ashwagandha root extract showed ~20% lifespan extension in a nematode model *Caenorhabditis elegans* [10]. Withanolide, a bioactive constituent of Ashwagandha showed a 29.7% extension in the mean lifespan and regulated the insulin/IGF-1 signaling (IIS) pathway and neural activity in *C. elegans* [11]. In human HeLa cell lines, Ashwagandha root extract, tested at various concentrations, led to an enhancement in telomerase activity by ~45% at 10–50 μg (assessed by the Telomerase Rapid Amplification Protocol (TRAP) assay) [12]. Ashwagandha extract also exhibited anti-genotoxic effects against H_2_O_2_-induced DNA damage in human peripheral blood lymphocytes [13]. Thus, considering the promising achievements in longevity promotion through in vitro and in vivo models, Ashwagandha deserves to be investigated in various degenerative and adult onset health ailments, with more understanding on potential anti-ageing mechanisms.

Guduchi [*Tinospora cordifolia* (Wild) Hook. f. & Thomson] is a celebrated *Rasayana* herb of Ayurveda. It is used at several dosage forms to treat inflammation, arthritis, allergy, diabetes and as an anti-ageing and rejuvenating tonic [14,15,16]. A study found that extracts from Guduchi markedly enhanced the rate of cell survival and protected against radiation-induced cytotoxicity and DNA damage in PC12 cells [17]. Another study using ethanolic Guduchi stem extracts reported DNA protective ability on sodium arsenite-induced genotoxicity in lymphocytes from Swiss Albino mice using the comet assay [18].

Mandukaparni [*Centella asiatica* (L.) Urban] is another renowned Ayurvedic herb effectively used to improve memory and for rejuvenation in traditional practices. The activity of extracts from this plant has been increasingly investigated on telomerase activity. In a study, the authors found that Mandukaparni extract was able to trigger an almost nine-fold increase in telomerase activity compared to untreated human peripheral blood mononuclear cells [19]. Interestingly, in rodent models, treatment with Mandukaparni extract showed improvement in cognitive functions through improving mitochondrial and antioxidant gene expression in the brain and liver [20]. The plant extracts also have also been shown to promote wound healing (possibly attributed to the presence of triterpenoid saponins) via the facilitation of new skin cell growth, increasing skin tensile strength and resilience, and inhibiting bacterial growth [21]. Castasterone, a Mandukaparni leaf-derived phytoconstituent, was also able to inhibit H_2_O_2_-induced DNA damage in a single cell gel electrophoresis assay (comet assay) [22].

Brahmi [*Bacopa monnieri* (L.) Wettst. In Eng. & Prantl] is another Ayurvedic plant traditionally used as a nootropic and tonic agent. A study performed on Brahmi extracts reported an extraordinary adaptogenic potential and role in scavenging superoxide anion and hydroxyl radicals and in reducing H_2_O_2_-induced cytotoxicity and DNA damage in human fibroblast cells [23]. Additionally, in another study, Brahmi methanol extract also demonstrated a marked protective activity against H_2_O_2_-induced cytotoxicity and DNA damage in human non-immortalized fibroblasts [24]. Furthermore, another investigation reported a significant antioxidant and DNA damage preventive effect (using pRSETA plasmid grown in *E. coli*) in such extracts [25]. In a further investigation, Brahmi extracts displayed protective effects against sodium nitroprusside (SNP)-induced DNA damage [26]. For bacosides, bioactive constituents of Brahmi, remarkable potentialities have been reported in terms of scavenging free radicals and protecting neural cells from cytotoxicity and DNA damage in Alzheimer’s disease [27].

Shankhapushpi (*Convolvulus pluricaulis* Choisy) is another Indian traditional plant widely used for its effective nootropic effects [28,29]. A study evaluated the neuroprotective potential of Shankhapushpi ethanol extract, and it was found to possess antioxidant and anti-apoptotic properties and to protect from H_2_O_2_-induced cytotoxicity and plasmid DNA damage [30].

Yashtimadhu (*Glycyrrhiza glabra* L.), aka Mulethi or Jethimadhu in traditional practice, is rich in glycyrrhizin (a triterpene saponin), and its root extracts have been reported to increase DNA resistance from CdCl_2_-induced genetic and oxidative damages in human lymphocytes [31]. In vitro, such extracts also protected plasmid pBR322 DNA and microsomal membranes from γ-irradiation-induced strand breaks [32]. In another study, Yashtimadhu ethanol extract used at a concentration of 250 μg/mL, led to a ~33.56% increase in survival rate and 14.28% increase in lifespan in *C. elegans* model [33].

Vacha (*Acorus calamus* Linn.), is another Ayurvedic plant with potent antioxidant and cytoprotective abilities, being able to effectively protect DNA from γ-radiation-induced strand breaks and to enhance DNA repair process *in vitro* [34,35].

Tulsi (*Ocimum basilicum* L.) essential oil has been shown to raise the apparent telomeres length in cell culture and to downregulate the telomeric repeat binding factor 1 (TERF-1) telomere length suppressor [36]. Other authors found that bioactive compounds present in seed extracts from another Tulsi variety, i.e., *Ocimum tenuiflorum* L., exerted a prominent antioxidant potential and conferred DNA protection in a plasmid DNA pBR322 model [37].

Haridra (*Curcuma longa* L.) is also an extensively used medicinal herb and soul of Indian cuisine. Haridra aqueous extracts and its main constituent, curcumin, are found to be protective against lipid peroxide-induced DNA damage [38], twigs-dry leaves smoke condensate-induced DNA damage in calf thymus DNA and human peripheral lymphocytes [39], and fuel smoke condensate-induced DNA damage in human lymphocytes [40], although the mechanism of action has not yet been identified. A recent study in a mouse model with carboplatin-induced myelosuppression suggested that curcumin promotes the DNA repair pathway in bone marrow [41]. In addition, following the curcumin interaction with Kelch-like ECH-associated protein 1 (Keap 1), the nuclear factor E2-related factor 2 (Nrf2) is released, which regulates antioxidant enzymes, anti-inflammatory response proteins, and DNA repair enzymes [42]. In *Drosophila melanogaster* [43] and *C. elegans* [44] models, curcumin led to a 25.8% and 25.0% increase in mean lifespan, respectively.

Several polyherbal Ayurvedic formulations are also being investigated for anti-ageing purposes. Amalaki Rasayana (AR), prepared from Amalaki (*Emblica officinalis* Gaertn.) fruits, is a time-tested Ayurvedic Rasayana drug, widely used for the prevention or even treatment of various age-related health conditions. AR markedly reduces the DNA damage in brain cells and confers genomic stability in neurons and astrocytes [45], and at same time raising the median lifespan and starvation resistance in *D. melanogaster* model [46]. AR has also been revealed to be able to suppress neurodegeneration in fly models of Huntington’s and AD [47]. A recent study with humans aged 45 to 60 years reported an increase in telomerase activity with no discernible change in telomere length in peripheral blood mononuclear cells following AR administration, suggesting that AR can avoid the telomeres erosion, promoting healthy ageing [48]. In aged human participants, AR intake maintained, or even enhanced, the DNA strand break repair, with no toxic effects [49]. Amalaki extract also exhibited neuroprotective effects from H_2_O_2_-induced DNA damage and repair in neuroblastoma cells [50].

Medhya Rasayana, a memory enhancer formulation prepared from a mixture of selected plants and their extracts, has a great ability to promote brain rejuvenation, triggering a marginal but sustained increase in constitutive DNA base excision repair in brain tissues of adult rats [51].

Another preparation, Brahma Rasayana, is a health-promoting formulation with >35 ingredients (*E. officinalis* and *Terminalia chebula* Retz. are the two major), increased constitutive DNA base excision repair and reduced clastogenicity [52].

Chyawanprash is also a popular health supplement traditionally used for rejuvenation, and displays cytoprotective and genoprotective effects [53], though more evidence is required to reinforce its longevity claims related to parameters, such as telomerase activation or telomere lengthening.

Triphala, a preparation of fruits of *Amalaki* (*E. officinalis*), *Bibhitaki* [*Terminalia bellerica* (Gaertn) Roxb.], and *Haritaki* (*T. chebula*), has shown a great ability to prevent and reverse radiation-induced DNA damage in various in vitro and animal models [54].

In short, the multiple Rasayana medicines reported in the Ayurveda literature, while extremely rich sources of key bioactive molecules, such as flavonoids and polyphenols, with remarkable antioxidant, adaptogenic, immunomodulatory, immunostimulant, cytoprotective and rejuvenating properties [8,9], underlines the hope that the ancient literary and experience-based knowledge base of Ayurveda has huge therapeutic potential, and thus can be used to discover and develop new anti-ageing drug candidates with potent telomerase activator, telomer protective and DNA repair properties.

## Figures and Tables

**Figure 1:**
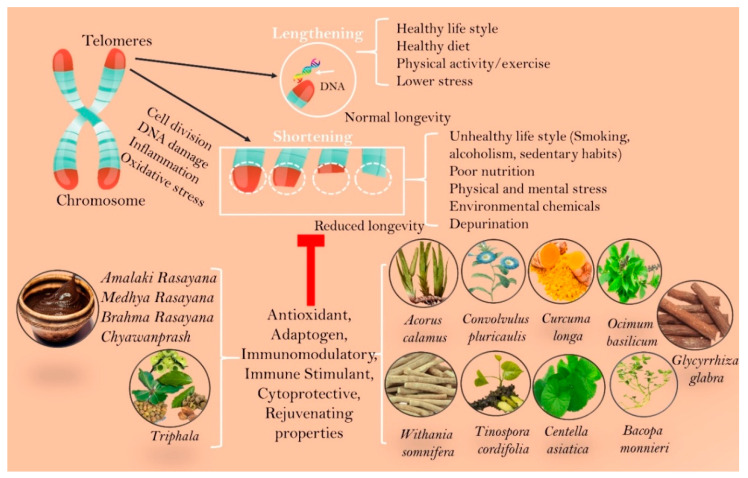
Potential anti-ageing Ayurveda medicines with telomer protective and DNA repair effects.
